# Strengthening Neglected Tropical Disease Research through Enhancing Research-Site Capacity: An Evaluation of a Novel Web Application to Facilitate Research Collaborations

**DOI:** 10.1371/journal.pntd.0003225

**Published:** 2014-11-13

**Authors:** Tamzin Furtado, Samuel Franzen, Francois van Loggerenberg, Gwenaelle Carn, Shannon Grahek, Megan McBride, Maureen Power, Jennifer O'Reilly, Barbara Savarese, Margaret Ann Snowden, Gwynn Stevens, Almarie Uys, Trudie Lang

**Affiliations:** 1 The Global Health Network, Centre for Tropical Medicine and Global Health, Nuffield Department of Clinical Medicine, University of Oxford, Oxford, United Kingdom; 2 Drugs for Neglected Disease Initiative (DNDi), Geneva, Switzerland; 3 Department of Microbiology, Immunology and Tropical Medicine, The George Washington University School of Medicine and Health Sciences, Washington, D.C., United States of America; 4 International Aids Vaccine Initiative (IAVI), New York, New York, United States of America; 5 Clinical Unit, PATH Malaria Vaccine Initiative, Washington, D.C., United States of America; 6 AERAS Global TB Vaccine Foundation, Washington, D.C., United States of America; 7 Global Alliance for TB Drug Development, Pretoria, South Africa; Instituto de Investigaciones en Enfrmedades Tropicales. Universidad Nacional de Salta, Argentina

## Background

The increasing number of clinical trials in developing countries brings unique challenges, including highly customised research infrastructure and narrow research focus. Many research sites have been equipped for one specific trial or disease area, often for a particular sponsor, with little consideration to subsequent diversification or sustainability [1]–[4]. Furthermore, the diseases studied in developing countries are seven to eight times more likely to be of relevance to developed countries than to developing countries [5],[6]. Accordingly, research sites are often only equipped to carry out research on diseases that are most relevant to developed nations—for example, noncommunicable diseases—rather than addressing local priority issues [7], [8].

There is further discrepancy between the amount of research conducted on neglected tropical tiseases (NTDs) when compared with “the big three”: human immunodeficiency virus (HIV), malaria, and tuberculosis (TB) [2]. One 2013 report estimates that only 1% of global spending in research and development is related to NTDs [5].

To foster research in NTDs and local disease priorities, it is important that research sites equip themselves with the skills, confidence, and knowledge to lead locally relevant research, not just in the conduct of clinical trials for the registration of new drugs and vaccines, but also in diagnostics, surveillance, disease management, and implementation science. Increasing the scope and diversity of investigations undertaken by research sites would contribute substantially to increasing the evidence base for diseases most relevant to developing countries, particularly NTDs [9].

Many partnerships aim to pool resources and support researchers by equipping them with the skills and knowledge to initiate their own research, including the World Health Organization's Tropical Disease Research Fellowship Scheme (WHO-TDR), European and Developing Countries Clinical Trials Partnership's (EDCTP's) Networks of Excellence, and free online resources such as the Global Health Network (www.theglobalhealthnetwork.org) [10], [11].

However, as reported by one eminent Central African researcher, there is still a gap in terms of enabling local researchers to make themselves known to other researchers: “There is no system or database where you can actually go and say, ‘In [my country], we can get this number of toxicologists and they are in these institutions or in this establishment.’…In Africa, people don't know each other exist and so cannot maximise resources and cannot work together.” When asked about how such problems could be overcome, numerous sites reported the need for an online tool that would allow them to highlight their experience for potential collaborators. For example, one responder stated that they thought “having an online database would be a great way of finding collaborators,” and another response indicated a need for “a platform to collaborate with stakeholders; [and] wider dissemination of opportunities.” No such tool or platform has previously existed, meaning that the skills and knowledge of research sites are effectively isolated.

A related frustration reported by research sites after a project concludes is the inability to find new studies to work on, meaning that sites often lose their trained staff as soon as their study-specific funding ends. This lack of access to new research collaborations is a major obstacle for those aiming to follow a career in research, as well as for sites, which frequently lose the capacity they have built up. Increasing the visibility of these sites will promote greater site sustainability and long-term support for research staff through improving access to diverse research experiences. The stability and accumulated knowledge, confidence, and training will ultimately help researchers improve site infrastructure and skill bases, thus enabling them to initiate their own research into local health issues [4], [5], [10], [12]. For example, strengthening the capacity of Africa-owned clinical trials to allow countries to pursue their own research and development (R&D) agendas has been identified as an international priority by WHO [4], [12]–[14].

Interestingly, sponsors and funders also experience difficulties in locating research sites with which to work. There is no publicly available global directory of sites or easy means of locating new partners. As a result, they often collaborate with the same research sites for each disease area, furthering this lack of diversification at research sites [14].

Against this background, a clear need emerged for a formal mechanism allowing established research sites and interested new researchers to promote their skills, facilities, staff, and experience to those planning studies. In response to this need, the Global Health Network has provided a resource called Site-Finder, a free and open-access online application. Adapting technology from dating websites, the application allows research sites to have an online presence and automatically suggests research collaborations between research sites and new research studies that have been posted on the site. The application has now been built, pilot-tested, and launched, and it is running successfully.

## Methods

Site-Finder was developed in 2012 by the Global Health Network, in collaboration with a working group comprising 13 members from the Bill and Melinda Gates Global Health Clinical Consortium, a collaboration of product development partnerships (PDPs) working in various areas of neglected tropical diseases and diseases of poverty research. The application was built and prepiloted on a staging site with the PDPs and eight research sites. Following revisions, a second iteration was released online in pilot format in November 2012, and research sites and groups planning studies were invited to sign up. Monitoring through constant online technical support was performed to identify and rectify any issues, and the final version was launched in June 2013.

After three months of online usage, an interim evaluation was performed in which registered research sites were sent a nine-item questionnaire via email. The questionnaire used a mixture of open-ended questions and rating scales to assess the ease of use, concept, need, and technical support of the Site-Finder application. Site-Finder was released formally in July 2013 and has been functioning successfully since then.

## Results

Feedback from sites responding to the interim evaluation (n = 16/50 or 32% of sites) was positive: 100% of users marked the concept of Site-Finder as “very good” or “good,” 81.3% of users rated Site-Finder “easy” or “very easy” to use, and 90% marked that they felt Site-Finder provided adequate support to those entering information about their sites

In terms of motivation, all sites that responded to the questionnaire mentioned wanting to find additional research collaborations for their sites, with one also wanting to share its own experiences with other sites.

**Figure 1:**
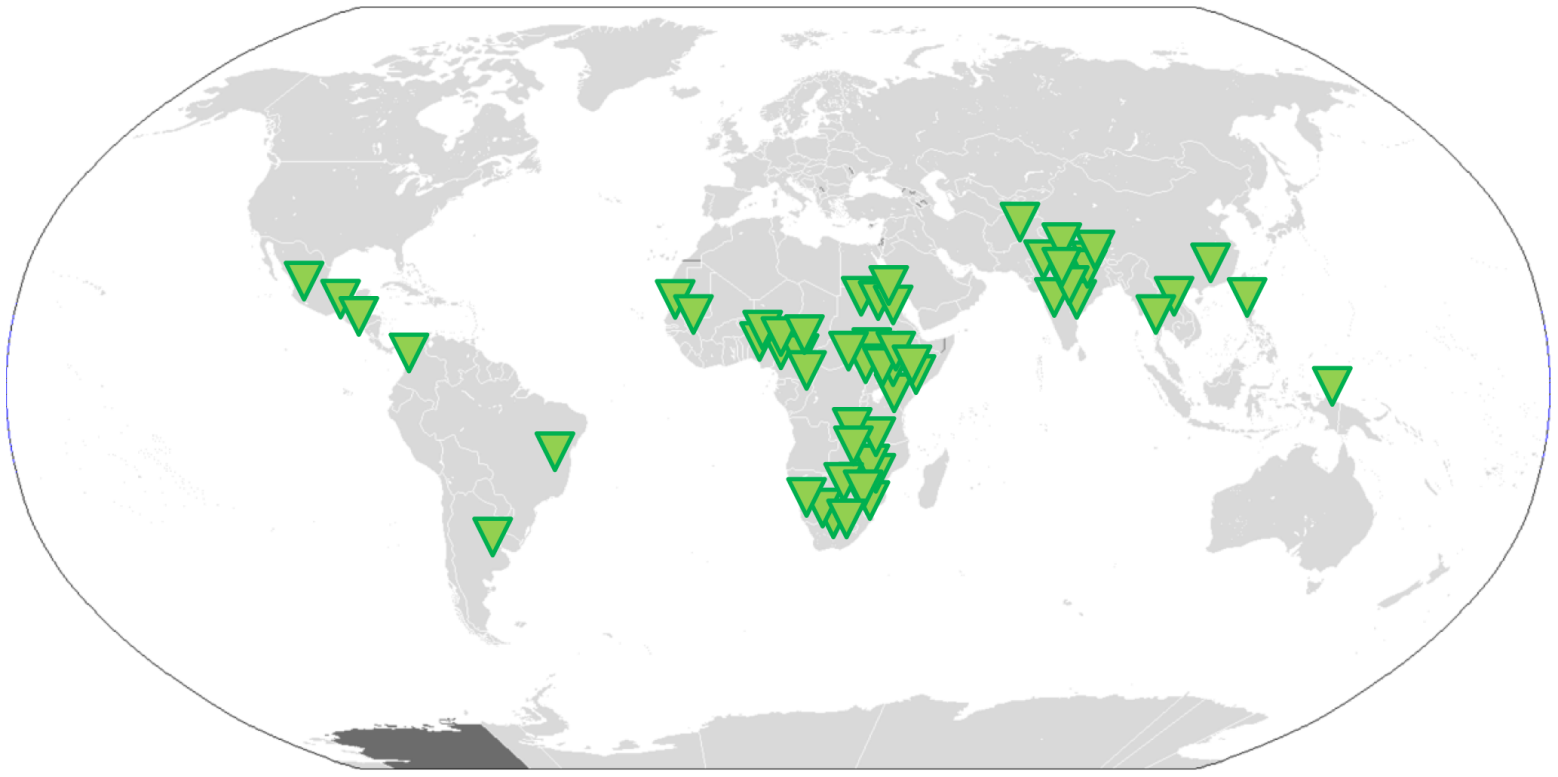
Map showing the distribution of 110 research sites as of July 2014.

During the six-month pilot phase, 52 research sites across 19 developing countries registered with Site-Finder, along with ten research projects seeking sites. The research sites represented great diversity in size and type, including large-scale national research institutes as well as small investigator-led sites at hospitals. Similarly, good diversity was shown in the research studies added: a mix of noncommercial organisations or universities and a range of disease areas and study types. Site-Finder continues to grow post–pilot phase, having received over 6,300 visits from over 3,800 users, across 142 countries (3 June 2014). Twenty studies and 110 research sites have registered (see Figure 1), and user reports of sites contacting one another and of studies making contact with new sites have come: in the past six months, over 35 “contact requests” have been made to sites that are part of Site-Finder as a result of their being on the platform.

Furthermore, we have received reports from sites stating other perceived benefits of being a part of Site-Finder, including receiving monthly news about global grant and funding opportunities, and making use of the other resources across the Global Health Network. For example, one site commented: “I particularly used the [research ethics eLearning] course for my MSc students very successfully. In addition we have been in contact with a number of sites on possible collaboration for a number of activities”; another commented, “I have benefited a lot from the posting of the grant calls.”

## Discussion

The feedback related to Site-Finder has been encouraging, with sites responding positively to the concept and need for this tool. Following Site-Finder's launch, the collaborative aim is that registered research sites will partner on more diverse research projects identified through Site-Finder and that this diversification will then allow sites greater sustainability and stability for staff with long-term broad training. We hope this will lead to a community of research sites with strong experience of conducting high-quality studies. These sites will then have the breadth of experience and independent capacity to initiate their own studies and engage in local research.

**Figure 2:**
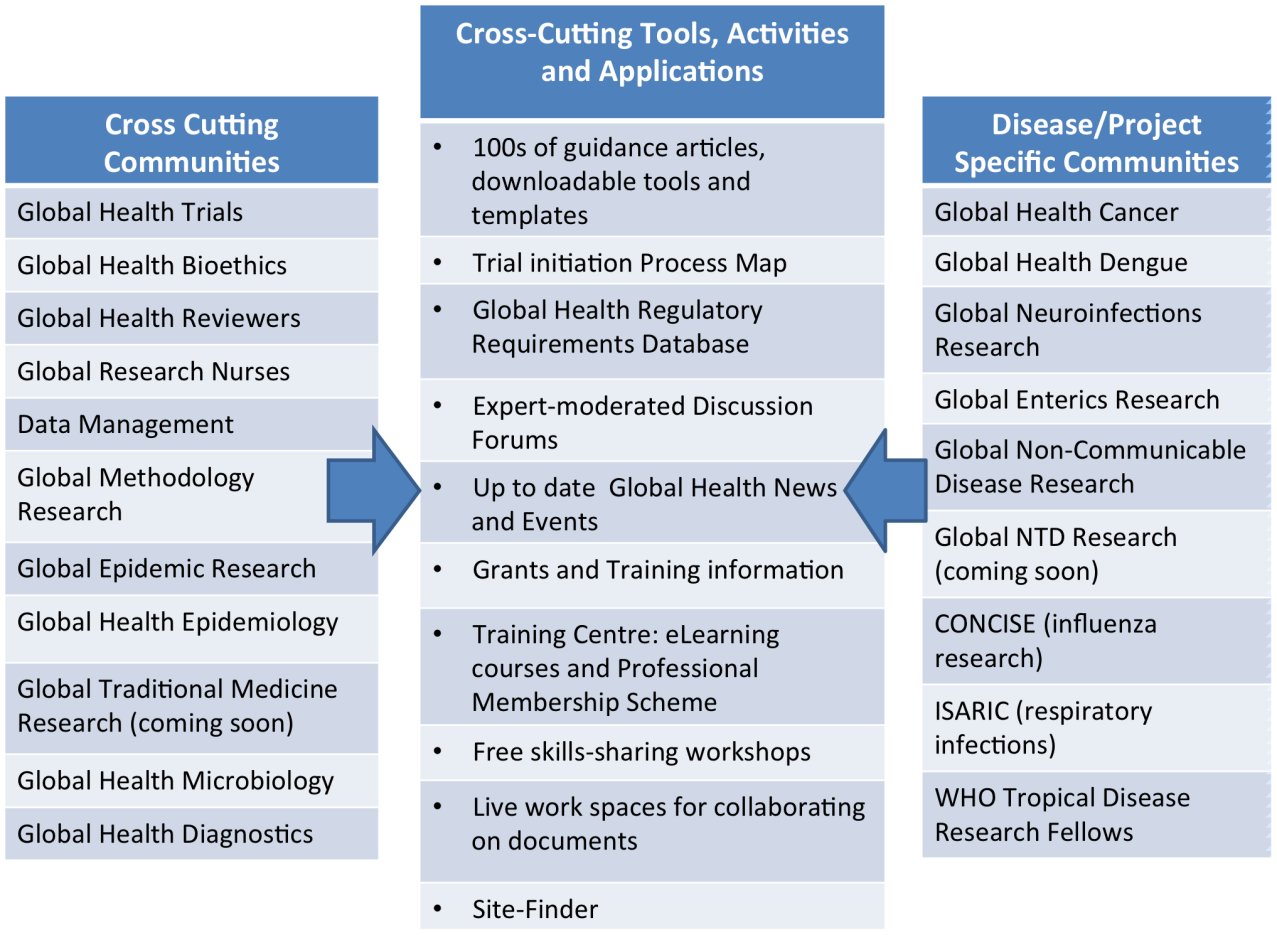
Applications and communities within the Global Health Network.

Situating Site-Finder within the Global Health Network, an existing, widely used community of researchers working in global health, provides distinct advantages. A single sign-on across the entire network means that one Site-Finder registration opens a further wealth of information to users who wish to further develop research skills and initiate their own studies. Such resources include regulatory information, guidance articles, downloadable tools and templates, eLearning courses, a Professional Membership Scheme to track continued professional development, and expert-moderated discussion forums (see Figure 2).

The Global Health Network aims to accelerate and streamline research through an innovative digital platform, facilitating collaboration and resource sharing in global health, and has attracted over 200,000 visits to date.

The Global Health Network is also keen to encourage sites to use Site-Finder in other ways, for example, by finding other local sites for shared training days, facilities, local research studies, or organised staff exchanges. Some localised and administrative support will be offered if sites want to participate in these activities.

The team will also be investigating ways of using Site-Finder to increase locally led research, for example, helping users of Site-Finder to access the other resources available on the Global Health Network for activities such as protocol writing, training staff, and reporting their work. One ongoing challenge faced by Site-Finder is whether it can support and encourage more locally relevant research or whether it will exacerbate the trend of encouraging studies mainly relevant to high-income locations [5]. So far, all the studies entered have been from noncommercial organisations or universities and relate mainly to relevant diseases, including NTDs, maternal health, HIV, and TB.

This concern was also discussed in detail within the working group that developed Site-Finder, and it was agreed that Site-Finder should allow commercial research opportunities to be posted because there is much value in facilitating different types of research at sites. The experience, funding, and training opportunities that arise from taking part in industry and other regulatory-type clinical trials are of enormous value to the sites involved. Furthermore, it is up to the sites themselves and the countries to decide who they collaborate with and what types of trials they wish to take part in. The objective of Site-Finder is to increase opportunities for researchers and their study teams. Working on a variety of study types will benefit sites by diversifying and overlapping funding streams, thereby keeping funding long-term and consistent, allowing for the development of infrastructure and a robust team of research staff who have experience in varied types of studies and in different disease areas.

Site-Finder will be continually assessed to ascertain whether it is working successfully—that is, linking research sites with sponsors and collaborators. Analytics data will be used to show the numbers of visitors from each location who have accessed the site and the information that they are accessing. Short semiannual questionnaires will be sent to the sites and collaborators to assess contact between Site-Finder users and to gain information on whether the research sites are also carrying out locally led research.

## Conclusions

It is important that researchers and sites are supported in developing comprehensive and sustainable research skills that enable the initiation of locally devised and locally led research studies. This requires access to guidance, tools, and resources for thorough training and support [10]. Diversifying research performed at sites will help to equip staff with the confidence and skills to initiate research into local health issues, therefore helping to create evidence to alleviate local disease burdens, particularly in neglected tropical diseases [10]. Site-Finder has been built, evaluated, and launched to enable research sites to engage in multiple and diverse research collaborations and thus strengthen their capacity. Because it is situated within the infrastructure of the Global Health Network, Site-Finder also allows sites to build their skills and knowledge by accessing the information and peer support that is abundant on the Network.

Although at an early stage, initial feedback on Site-Finder is highly positive. This indicates that the web application will successfully link research sites and new projects and succeed in the goal of increasing equity in access to research opportunities.

## References

[1] Montaner JS, O'Shaughnessy MV, Schechter MT (2001) Industry-sponsored clinical research: a double-edged sword. Lancet 358: 1893–1895. 11741648 10.1016/S0140-6736(01)06891-X

[2] Glickman SW, McHutchison JG, Peterson ED, Cairns CB, Harrington RA, et al (2009) Ethical and scientific implications of the globalization of clinical research. N Engl J Med 360: 816–823. 19228627 10.1056/NEJMsb0803929

[3] Global Network for Neglected Tropical Diseases (2005) Neglected tropical diseases and the post-2015 agenda: addressing a cross-cutting development issue that improves the lives of marginalized populations. Available: http://www.globalnetwork.org/sites/default/files/Neglected%20Tropical%20Diseases%20and%20the%20Post-2015%20Development%20Agenda%202013.pdf. Accessed 8 October 2014.

[4] Ravinetto RM, Talisuna A, De Crop M, van Loen H, Menten J, et al (2013) Challenges of non-commercial multicentre North-South collaborative clinical trials. Trop Med Int Health 18: 237–241. 23217117 10.1111/tmi.12036

[5] Rottingen JA, Regmi S, Eide M, Young AJ, Viergever RF, et al (2013) Mapping of available health research and development data: What's there, what's missing, and what role is there for a global observatory? Lancet 382: 1286–1307. 23697824 10.1016/S0140-6736(13)61046-6

[6] World Health Organization (2011) Accelerating Work to Overcome the Impact of Neglected Tropical Diseases: A Roadmap for Implementation. Geneva, Switzerland: World Health Organization. Available: http://whqlibdoc.who.int/hq/2012/WHO_HTM_NTD_2012.1_eng.pdf. Accessed 8 October 2014.

[7] Costello A, Zumla A (2000) Moving to research partnerships in developing countries. BMJ 321: 827–829. 11009530 10.1136/bmj.321.7264.827 PMC1118627

[8] Trouiller P, Olliaro P, Torreele E, Orbinski J, Laing R, et al (2002) Drug development for neglected diseases: a deficient market and a public-health policy failure. Lancet 359: 2188–2194. 12090998 10.1016/S0140-6736(02)09096-7

[9] Lang TA, White NJ, Tran HT, Farrar JJ, Day NP, et al (2010) Clinical research in resource-limited settings: enhancing research capacity and working together to make trials less complicated. PLoS Negl Trop Dis 4: e619. 20614013 10.1371/journal.pntd.0000619 PMC2894123

[10] Franzen SR, Chandler C, Enquselassie F, Siribaddana S, Atashili J, et al (2013) Understanding the investigators: A qualitative study investigating the barriers and enablers to the implementation of local investigator-initiated clinical trials in Ethiopia. BMJ Open 3: e003616. 10.1136/bmjopen-2013-003616 PMC3845054 24285629

[11] Lang T (2011) Advancing global health research through digital technology and sharing data. Science 331: 714–717. 21311011 10.1126/science.1199349

[12] Tinto H, Noor RA, Wanga CL, Valea I, Mbaye MN, et al (2013) Good clinical practice in resource-limited settings: translating theory into practice. Am J Trop Med Hyg 88: 608–613. 23553224 10.4269/ajtmh.12-0330 PMC3617841

[13] Matsoso P, Auton M, Banoo S, Fomundham H, Leng H, et al. (2005) How does the regulatory framework affect incentives for research and development? A proposal for a regulatory framework to improve regulatory capacity and introduce incentives for research and development in areas of public health importance. 2005, Geneva, Switzerland: World Health Organization. Available: http://www.who.int/intellectualproperty/studies/Study5.pdf?ua=1. Accessed 8 October 2014.

[14] The PLoS Medicine Editors (2004) A new vision for clinical trials in Africa. PLoS Med 1: e71. 15630472 10.1371/journal.pmed.0010071 PMC539056

